# Two nanoformulations induce reactive oxygen species and immunogenetic cell death for synergistic chemo-immunotherapy eradicating colorectal cancer and hepatocellular carcinoma

**DOI:** 10.1186/s12943-020-01297-0

**Published:** 2021-01-06

**Authors:** Jianfeng Guo, Zhuo Yu, Dandan Sun, Yifang Zou, Yun Liu, Leaf Huang

**Affiliations:** 1 School of Pharmaceutical Sciences, Jilin University, Changchun, 130021 China; 2 Division of Pharmacoengineering and Molecular Pharmaceutics, Eshelman School of Pharmacy, University of North Carolina, Chapel Hill, NC 27599 USA; 3 Department of Hepatopathy, Shuguang Hospital, Affiliated to Shanghai University of Traditional Chinese Medicine, Shanghai, 201203 China

**Keywords:** Nanoparticles, Drug delivery, Immunogenic cell death, Reactive oxygen species, Combination therapy

## Abstract

**Background:**

FOLFOX is a combinational regimen of folinic acid (FnA, **FOL**), fluorouracil (5-Fu, **F**) and oxaliplatin (OxP, **OX**), and has been long considered as the standard treatment of colorectal cancer (CRC) and hepatocellular carcinoma (HCC). Recent developments of nano delivery systems have provided profound promise for improving anticancer efficacy and alleviating side effects of FOLFOX. Previously, a nanoformulation (termed Nano-Folox) containing OxP derivative and FnA was developed in our laboratory using nanoprecipitation technique. Nano-Folox induced OxP-mediated immunogenic cell death (ICD)-associated antitumor immunity, which significantly suppressed tumor growth in the orthotopic CRC mouse model when administrated in combination with free 5-Fu.

**Methods:**

A nanoformulation (termed Nano-FdUMP) containing FdUMP (5-Fu active metabolite) was newly developed using nanoprecipitation technique and used in combination with Nano-Folox for CRC and HCC therapies.

**Results:**

Synergistic efficacy was achieved in orthotopic CRC and HCC mouse models. It resulted mainly from the fact that Nano-FdUMP mediated the formation of reactive oxygen species (ROS), which promoted the efficacy of ICD elicited by Nano-Folox. In addition, combination of Nano-Folox/Nano-FdUMP and anti-PD-L1 antibody significantly inhibited CRC liver metastasis, leading to long-term survival in mice.

**Conclusion:**

This study provides proof of concept that combination of two nano delivery systems can result in successful FOLFOX-associated CRC and HCC therapies. Further optimization in terms of dosing and timing will enhance clinical potential of this combination strategy for patients.

**Graphical abstract:**

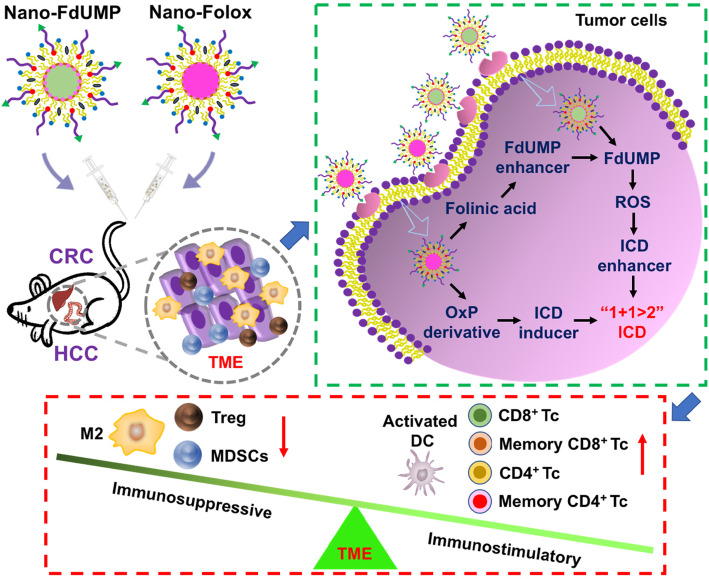

**Supplementary Information:**

The online version contains supplementary material available at 10.1186/s12943-020-01297-0.

## Introduction

The FOLFOX regimen including folinic acid (FnA), fluorouracil (5-Fu) and oxaliplatin (OxP) has been used as the standard chemotherapy for patients with colorectal cancer (CRC) and hepatocellular carcinoma (HCC) at advanced stages [1, 2]. FOLFOX has also provided therapeutic benefits for patients with unresectable CRC liver metastases [3]. The clinical practice of FOLFOX includes: 1) patients are intravenously (i.v.) infused with OxP and FnA simultaneously, which are followed by i.v. administration of 5-Fu; 2) patients receive FOLFOX for 8 to 12 cycles every 2 to 3 weeks. Once inside tumor cells, the activity of 5-Fu is enhanced by FnA, and 5-Fu/FnA adds or synergizes with OxP [1, 2]. Notably, major barriers associated with FOLFOX including non-specific delivery, high toxicity and long course of treatment still limits its clinical application [4, 5]. It is widely established that nanomaterials may be designed for tissue- and cell-specific delivery of chemotherapeutics, which will improve therapeutic efficacy against tumors and reduce damage to healthy tissues [6]. Therefore, development of nano delivery systems holds great promise for overcoming the barriers associated with FOLFOX.

We have recently produced a nanoscale precipitate (C_26_H_35_N_9_O_7_Pt) using the adduct of [Pt (DACH)(H_2_O)_2_]^2+^ (active form of OxP) and FnA^2−^ [7]. C_26_H_35_N_9_O_7_Pt could be encapsulated into an aminoethyl anisamide (AEAA)-targeted polyethylene glycol (PEG)-modified lipid nanoparticle (NP) using nanoprecipitation process, forming a nanoformulation termed Nano-Folox [7]. AEAA is a high affinity (Kd ≈ 9 nM) ligand for the sigma-1 receptor which is overexpressed in most of solid tumor cells [8–11]. Nano-Folox induced robust anticancer immunity, which is mainly due to OxP-mediated immunogenic cell death (ICD) [12]. When administrated in combination with free 5-Fu, Nano-Folox at lower doses led to significantly enhanced tumor regression in an orthotopic CRC mouse model than FOLFOX, i.e., free drugs in combination, at higher doses. Moreover, a combination strategy “Nano-Folox/5-Fu and anti-PD-L1 monoclonal antibody (mAb)” remarkably slowed down CRC liver metastasis. It is worth noting that although tumor growth was significantly inhibited during treatment period, tumors progressed rapidly after dosing, and no long-term survival was achieved in mice. These results imply that tumor recurrence is caused most likely due to the lack of tumor-specific memory immune response.

It has been reported that the differentiation of naïve T cells is highly associated with the antigen availability to DCs [13], and higher amount and longer duration of antigen stimulation produce larger number of effector and memory T cells [14]. ICD can induce the exposure of damage-associated molecular patterns (DAMPs) from dying or dead cancer cells, resulting in the antigen presentation to DCs for tumor-specific T cell response [15]. It has also been reported that the induction of ICD is accompanied with the formation of reactive oxygen species (ROS) [15], and the ICD efficacy may be enhanced by ROS-inducing strategies [16–18]. Therefore, we hypothesize that the ROS induction may be safely and effectively achieved by targeted delivery of 5-Fu using nano delivery systems, which will synergize with Nano-Folox to induce effector and memory T cells for tumor-specific killing and protective responses.

Consequently, an AEAA-targeted PEGylated lipid NP (termed Nano-FdUMP) was newly produced using nanoprecipitation technique for delivery of 5-Fluoro-2′-deoxyuridine 5′-monophosphate (FdUMP, an active 5-Fu metabolite) [19]. The chemo-immunotherapeutic efficacy of Nano-FdUMP was investigated using orthotopic and metastatic mouse models when applied alone or in combination with Nano-Folox and anti-PD-L1 mAb.

## Methods

### Materials

5-Fluoro-2′-deoxyuridine 5′-monophosphate (FdUMP), 2′-deoxyuridine 5′-monophosphate (dUMP), dichloro (1,2-diaminocyclohexane) platinum (II), AgNO_3_, IGEPAL® CO-520, cyclohexane, Triton X-100, CaCl_2_, (NH_4_)_2_HPO_4_, cholesterol, folinic acid (FnA) and 5-Fluorouracil (5-Fu) were obtained from Sigma-Aldrich. Oxaliplatin (OxP) was obtained from Selleckchem. 1,2-dioleoyl-*sn*-glycero-3-phosphate (DOPA) and 1,2-dioleoyl-3-trimethylammonium-propane (DOTAP) were purchased from Avanti Polar Lipids. N-(Carbonyl-methoxypolyethyleneglycol 2000)-1,2-distearoyl-sn-glycero-3-phosphoethanolamine (SUNBRIGHT® DSPE-020CN; DSPE-PEG) was obtained from NOF CORP. DSPE-PEG-AEAA was synthesized as previously demonstrated by our laboratory [20].

### Preparation and characterization of nanoformulations

Nano-FdUMP was prepared as previously described with modifications [21, 22]. Briefly, 1 mL of FdUMP solution (1 mg/mL) was added into 2 mL of CaCl_2_ solution (2.5 M), and this mixture was added into 80 mL oil phase composed of IGEPAL® CO-520 and cyclohexane (30:70, V:V) for the generation of water-in-oil reverse microemulsion. Another microemulsion (80 mL) was prepared by adding 2 mL of (NH_4_)_2_HPO_4_ solution (50 mM) and 1 mL of DOPA solution (20 mM in chloroform). Two microemulsions were stirred for ~ 15 to 20 min. After this, 160 mL of ethanol were added for ~ 15 to 20 min with stirring, which was followed by centrifugation for ~ 20 min at 10,000 g for collection of nanoprecipitates. Nanoprecipitates were washed using ethanol, dried using nitrogen, and stored in chloroform.

The optimal ratio between nanoprecipitates and outer leaflet lipids for Nano-FdUMP was as follows: 1500 μg of nanoprecipitates, 30 μL of DOTAP (25 mM), 30 μL cholesterol (25 mM) and 20 μL DSPE-PEG/DSPE-PEG-AEAA (20 mM, molar ratio = 5:1) in 2 mL of chloroform. This theoretically achieved ~ 3.5 mol% of AEAA on the outer lipid surface per formulation. Following the evaporation of chloroform, the lipid film was resuspended to form Nano-FdUMP aqueous solution. The encapsulation efficiency and loading capacity were assessed using HPLC (Shimadzu, Japan) (C18 column, UV at 250 nm, mobile phase = water and methanol, 85:15). Nano-dUMP and non-targeted Nano-FdUMP were prepared as mentioned above except the use of dUMP and the lack of DSPE-PEG-AEAA, respectively.

In addition, Nano-Folox was prepared as previously described [7]. Briefly, AgNO_3_ (64.5 mg, 0.38 mmol) was added with dichloro (1,2-diaminocyclohexane) platinum (II) (76 mg, 0.2 mmol) in 1 mL deionized water, in order to produce the dihydrate (1,2-diaminocyclohexane) platinum (II). After this, the solution was heated at ~ 60 °C for 3 h and continued with stirring at RT overnight. Subsequently, the concentration of dihydrate (1,2-diaminocyclohexane) platinum (II) was measured using inductively coupled plasma mass spectrometry (ICP-MS) [23]. 100 μL dihydrate (1,2-diaminocyclohexane) platinum (II) (100 mM) were added into a 25 mL oil phase (hexanol, Triton X-100 and cyclohexane; 10:15:75, V:V:V) for the production of water-in-oil reverse microemulsion. Moreover, 2 mL FnA (10 mM) and 200 μL DOPA (20 mM) were added into a 75 mL oil phase with stirring to produce another water-in-oil reverse microemulsion. After 15 min, two microemulsions were mixed for ~ 45 min with stirring. Subsequently, 100 mL ethanol were added with stirring for ~ 15 min, which were followed by the centrifugation for ~ 20 min at 10,000 g to collect nanoprecipitates (C_26_H_35_N_9_O_7_Pt). For 1 mg nanoprecipitate core, 10 μL of 20 mM DOTAP, 10 μL of 20 mM cholesterol and 5 μL 20 mM DSPE-PEG/DSPE-PEG-AEAA (molar ratio = 4:1) were added into 1 mL chloroform. After evaporating chloroform, the lipid film was reconstituted using deionized water to form Nano-Folox.

The hydrodynamic diameter and zeta potential of NPs were measured using Malvern Nano-ZS. The morphology of NPs was observed using the JEM1230 (JEOL) transmission electron microscope (TEM). In addition, a solution of NPs with 200 μg of FdUMP was incubated at 37 °C in 0.01 M PBS (pH = 5.5 and 7.4) with shaking. Samples were obtained at different time points for centrifugation at 10,000 g for ~ 30 min. The concentration of free FdUMP within supernatants (dissociated from nanoprecipitates) was determined using HPLC.

### Cell culture

CT26 (mouse CRC cell line), Hepa1–6 (mouse HCC cell line), 4 T1 (mouse breast cancer cell line) and B16 (mouse melanoma cell line) cells were cultured using DMEM (Gibco) with 10% bovine calf serum (Hyclone) and 1% antibiotic-antimycotic (Gibco). CT26-FL3 (a subtype of CT26, it is engineered to stably express luciferase) and Hepa1–6-Luc (it is engineered to stably express luciferase) cells [7, 24] were cultured using the aforementioned growth medium with 1 μg/mL puromycin (ThermoFisher). Cells were maintained at 37 °C with 5% CO_2_ and 95% relative humidity.

### In vitro studies

MTT assay was applied to determine in vitro cytotoxicity. CT26 and Hepa1–6 cells (1 × 10^4^/well) were cultured within 96-well plates, respectively. Following one day incubation, 5-Fu, Nano-dUMP and Nano-FdUMP were added to cells for 24 h. Cells were then added with MTT reagent at 37 °C for ~ 4 h before measurement at 570 nm. IC_50_ was calculated using the GraphPad Prism software.

CT26 and Hepa1–6 cells (5 × 10^4^/well) were placed into 24-well plates, respectively. After one day incubation, cells were treated with or without N-acetylcysteine (NAC; 5 mM) for 4 h. Cells were replaced with fresh growth medium and added with 5-Fu, Nano-dUMP and Nano-FdUMP (all at 15 μM) for 24 h. Subsequently, apoptotic cells were detected using Annexin V-FITC/propidium iodide assay (Promega) and measured by the Becton Dickinson FACSCalibur. In a separate experiment, the ROS level in cells was detected using 2′,7′-dichlorodihydrofluorescein diacetate-based Reactive Oxygen Species Assay Kit (YIASEN Biotech) by microplate reader (488 nm/525 nm).

The CRT exposure was detected using immunofluorescence staining assay. CT26 and Hepa1–6 cells (60,000 per well) were cultured in 8-well chamber slides (ThermoFisher). Following one day incubation, cells were treated with or without NAC (5 mM) for 4 h. Cells were then replaced with fresh growth medium and treated with either Nano-FdUMP (15 μM), Nano-Folox (5 μM), or both (Nano-Folox was first added, and FdUMP was added at 2 h later; this sequential administration was same for in vitro studies unless mentioned otherwise). Two h post treatment, cells were incubated with 0.25% paraformaldehyde (PFA). Following 5 min incubation, cells were washed with PBS, which were followed by the application of anti-CRT antibody (ab2907, Abcam, 1:500) for 1 h. After PBS washes, FITC-conjugated secondary antibodies (ab150077, Abcam) were added into cells for 30 min. Subsequently, cells were treated by 4% PFA for 20 min and stained using DAPI (ThermoFisher) for confocal imaging (LSM-710, Zeiss).

In order to measure the extracellular ATP, CT26 and Hepa1–6 cells were placed into 24-well plates at a density of 60,000 cells per well. After one day incubation, cells were treated with or without NAC (5 mM) for 4 h. Cells were replaced with fresh growth medium and added with either Nano-FdUMP (15 μM), Nano-Folox (5 μM), or both for 24 h. Subsequently, extracellular ATP was detected using ENLITEN® ATP Assay System Bioluminescence Detection Kit.

The release of HMGB1 was analyzed using ELISA assay. CT26 and Hepa1–6 cells (60,000 per well) were cultured in 24-well plates. Following one day incubation, cells were treated with or without NAC (5 mM) for 4 h. Cells were replaced with fresh growth medium and added with either Nano-FdUMP (15 μM), Nano-Folox (5 μM), or both for 8 h. After this, the level of HMGB1 in the supernatants was measured using ELISA kit (LS-F11641, LifeSpan BioSciences).

### In vivo toxicity, pharmacokinetics and biodistribution

Six-week old female BALB/C and male C57BL/6 mice were purchased from Charles River Laboratories. The procedures used in this study were approved by Institutional Animal Care and Use Committee of University of North Carolina at Chapel Hill and by the Animal Ethics Committee of Jilin University.

Healthy mice were treated with nanoformulations as described in Figs. S2 and S9 (*n* = 5). Body weight was recorded, and the whole blood and the serum of animals were obtained on Day 35 to analyze myelosuppression and hepatic/renal functions.

The orthotopic CRC mouse model was achieved as previously described [7]. Briefly, BALB/C mice were anesthetized by 2.5% isoflurane, and the cecum wall was injected with ~ 1 × 10^6^ CT26-FL3 cells. In addition, the orthotopic HCC mice were established as previously described [25]. Briefly, C57BL/6 mice were anesthetized by 2.5% isoflurane, and the liver was injected with ~ 1 × 10^6^ Hepa1–6-Luc cells. Following tumor inoculation (Day 0), animals were intraperitoneally (i.p.) injected with 100 μL luciferin (10 mg/mL; Pierce™), and tumor growth was measured using IVIS® Kinetics Optical System (Perkin Elmer). When tumor growth was reached at ~ 0.5 to 1 × 10^9^ p/sec/cm^2^/sr, pharmacokinetics and tissue distribution studies were investigated as follows: 1) 5-Fu (10 mg/kg) or Nano-FdUMP containing 10 mg/kg of fluorine drug were i.v. administrated, and the blood (~ 50 μL) was collected at 1, 5, 10, and 15 min, and 0.5, 1, 4, 8 and 12 h (*n* = 4). As previously described [26], plasma samples were extracted with ethyl acetate, dried with nitrogen, and reconstituted in the mobile phase (water/methanol, 85:15). The concentration was assessed using HPLC (Shimadzu, Japan) (C18 column, UV at 265 nm for 5-Fu and UV at 250 nm for FdUMP). Half-life was evaluated using DAS 2.0 software. In separate studies, ~ 0.05 wt% of DiD (ThermoFisher) was formulated into Nano-FdUMP or non-targeted counterpart (10 mg/kg of fluorine drug). Twelve h post i.v. administration, distribution of DiD-labeled nanoformulations into tissues and tumors was detected (640 nm/670 nm) using IVIS® Kinetics Optical System (*n* = 4).

### Synergistic efficacy of nano-FdUMP and nano-Folox in orthotopic CRC and HCC mouse models

When tumor growth was reached at ~ 0.5 to 1 × 10^9^ p/sec/cm^2^/sr, tumor-bearing mice were injected with either OxP/FnA (1.5 mg/kg and 4.5 mg/kg, i.v.) or Nano-Folox containing 1.5 mg/kg of platinum drug (i.v.; due to the molar ratio of OxP derivative and FnA = 1:1 for nanoprecipitate (C_26_H_35_N_9_O_7_Pt) [7], it contained ~ 4.5 mg/kg of FnA) as described in figs. 5 and 6. Eight h post injection (t_1/2_ of Nano-Folox ≈ 1.4 h), tumor-bearing mice were treated with either 5-Fu (10 mg/kg; i.v.) or Nano-FdUMP containing 10 mg/kg of fluorine drug (i.v.). Tumor growth was observed using the IVIS® Kinetics Optical System (*n* = 6).

In separate experiments, 3 days after two injections (time point to analyze chemotherapeutic and immunotherapeutic effects was generally chosen within one week following treatment to ensure reliable analyses) [10, 27–29], tumors were obtained on Day 24 (CRC) and Day 23 (HCC) for following assays: 1) TUNEL assay [7, 24]. It was performed using the DeadEnd™ Fluorometric TUNEL System (Promega) (*n* = 4). DNA fragments (FITC) and nuclei (DAPI) were detected by confocal microscopy; 2) Flow cytometry [7, 24]. Tumors (*n* = 4) were treated using collagenase A (1 mg/mL; Sigma) and DNAse (200 μg/mL; Invitrogen) for 30 min at 37 °C to produce single cells. After lysis of erythrocytes with ACK buffer (Gibco), cells were treated by fluorophore-labeled antibodies (see Supplementary Table 1), fixed using 4% PFA, and assessed using the Becton Dickinson LSR II. 3) RT-PCR assay [7, 24]. Total RNA samples (n = 4) were obtained using the Qiagen RNeasy® Microarray Tissue Mini Kit. cDNA was generated by a BIO-RAD iScript™ cDNA Synthesis Kit. The RT-PCR reaction was carried out using the TaqMan Gene Expression Master Mix (BIO-RAD) by the 7500 Real-Time PCR System. The information of primers was shown in Supplementary Table 2.

The depletion study of CD4^+^ and CD8^+^ T cells was performed as previously described [7, 24]. In brief, 100 μg of either anti-CD8 (clone 53–6.72, Bioxcell), anti-CD4 (clone GK1.5, Bioxcell) or IgG (Bioxcell, polyclonal) antibodies were i.p. injected per mouse at respective schedules (figs. 5 and 6) before the treatment of Nano-FdUMP/Nano-Folox. Tumor growth was measured using the IVIS® Kinetics Optical System (*n* = 4).

### Combination therapy of nano-FdUMP and nano-Folox with PD-L1 blockade for CRC liver metastasis mouse model

The CRC liver metastasis mouse model was established as previously described [7]. In brief, mice were anesthetized using 2.5% isoflurane, and the spleen was exteriorized, tied and sectioned. Afterwards, ~ 2 × 10^5^ CT26-FL3 cells were injected to the distal section of the spleen. The hemi-spleen injected by CT26-FL3 cells was removed, and the other half was placed back into the cavity. Following tumor inoculation at Day 0, tumor growth was monitored using the IVIS® Kinetics Optical System. When tumor growth was reached at ~ 0.5 to 1 × 10^8^ p/sec/cm^2^/sr, mice were i.v. administrated with Nano-Folox containing 1.5 mg/kg of Pt (~ 4.5 mg/kg of FnA) as described in fig. 7, which were followed by i.v. administration of Nano-FdUMP (10 mg/kg of fluorine drug) at 8 h post-injection. After this, mice were i.p. injected with or without anti-PD-L1 mAb (Bioxcell, clone 10F.9G2, 100 μg per mouse). The tumor growth was observed using the IVIS® Kinetics Optical System (*n* = 6). Separately, one day following two injections, tumors were obtained on Day 12 for TUNEL analysis (*n* = 4), flow cytometry (n = 4) and RT-PCR experiment (n = 4), as described above.

### Statistical analysis

Data were presented in this work as mean ± standard deviation (SD). The significance between two groups was evaluated using unpaired Student’s t-test (two-tailed). The significance between three or more groups was assessed using the two-way ANOVA (Bonferroni’s Post-Hoc model). A log rank test was utilized for comparison in survival study. In this work, *p* < 0.05 was considered statistically significant.

## Results

### Preparation and physicochemical characterization of nano-FdUMP

**Fig. 1:**
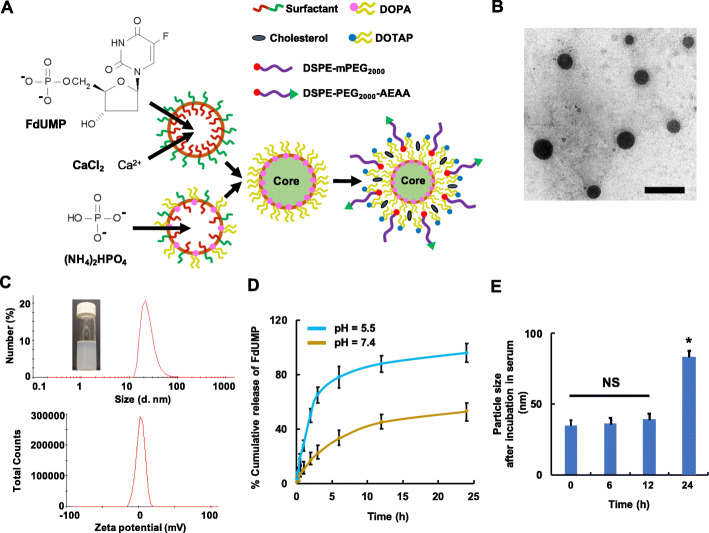
The preparation and physicochemical characterization of Nano-FdUMP. **a**) A schematic of Nano-FdUMP developed in microemulsions using nanoprecipitation technique. **b**) TEM image of Nano-FdUMP (bar = 100 nm). **c**) Size distribution (~ 35 nm, polydispersity index ≈ 0.3) and surface charge (~ 2 mV) of Nano-FdUMP. **d**) The in vitro release of fluorine drug from nanoprecipitates within Nano-FdUMP in pH = 5.5 and 7.4 (*n* = 4). **e**) The stability of Nano-FdUMP following the incubation of 10% serum-containing medium for 0, 6, 12 and 24 h at 37 °C

One water-in-oil microemulsion containing CaCl_2_ and FdUMP was mixed with another water-in-oil microemulsion containing Na_2_HPO_4_, in order to generate Ca_3_(PO_4_)_2_ amorphous precipitate in which FdUMP was entrapped (fig. 1a). The Ca_3_(PO_4_)_2_-FdUMP nanoprecipitate was stabilized by 1,2-dioleoyl-sn-glycero-3-phosphate (DOPA), and the stabilized nanoprecipitate was coated with 1,2-dioleoyl-3-trimethylammonium-propane (DOTAP), cholesterol, 1,2-distearoyl-sn-glycero-3-phosphoethanolamine-PEG_2000_ (DSPE-PEG) and DSPE-PEG-AEAA, resulting in Nano-FdUMP (fig. 1b). Nano-FdUMP is reminiscent of other nanoformulations containing Ca_3_(PO_4_)_2_-nucleic acid nanoprecipitate that have also been developed using nanoprecipitation process in our lab [21–23, 28, 30–34]. Nano-FdUMP illustrated nanoscale particle size (~ 35 nm, polydispersity index ≈ 0.3) and neutral surface charge (~ 2 mV) (fig. 1c). The encapsulation efficiency (EE %) and loading capacity (LC %) of FdUMP in Nano-FdUMP were ~ 98% and ~ 38 wt%, respectively, as measured using HPLC, which were similar to EE % and LC % for FdUMP in Nano-FdUMP without AEAA. As shown in fig. 1d, > 95% of FdUMP were released from the nanoprecipitate inside Nano-FdUMP when incubated within acidic PBS for 24 h, which was significantly more efficient than the drug release in neutral PBS. These indicate that Nano-FdUMP showed pH-sensitive drug release, which is most likely due to the acid-sensitive feature of Ca_3_(PO_4_)_2_ [35]. Nano-FdUMP maintained in vitro stability in serum-containing medium up to 12 h, but significant aggregation occurred at 24 h (fig. 1e). In addition, Nano-FdUMP without AEAA demonstrated similar morphology, particle size, surface charge, drug release and serum stability (Fig. S1) as observed for Nano-FdUMP (fig. 1).

Recently, several nanoformulations have been developed for delivery of 5-Fu in tumor-bearing mouse models [36–38]. For example, Li et al. produced a poly(γ-benzyl-L-glutamate)-based NP for delivery of 5-Fu in subcutaneous CRC mouse model; however, EE% and LC% were only ~ 61% and ~ 27%, respectively [36]. Safwat and colleagues also developed a gold NP-based system for delivery of 5-Fu in skin cancer mouse model, but EE% was less than 70% (LC% was not mentioned) [37]. In addition, Kazi and coworkers designed a poly (lactic-co-glycolic acid)-based NP for delivery of 5-Fu in melanoma mouse model, but EE% and LC% were only ~ 56% and ~ 2%, respectively [38]. In the present study, Nano-FdUMP achieved significantly higher EE% (~ 98%) and LC% (~ 38%) of fluorine drug than these previously reported studies.

It is known that 5-Fu can be metabolized into FdUMP within cancer cells, and FdUMP forms a complex with thymidylate synthase for inhibition of deoxythymidine monophosphate (dTMP) production [19]. However, intracellular metabolism of 5-Fu into FdUMP is a rate-limiting process that dampens therapeutic efficacy; for example, over 80% of a single dose of 5-Fu is converted to inactive metabolites [39]. In addition, although 5-Fu is well tolerated, serious toxic signs are found in patients who have deficiency of dihydropyrimidine dehydrogenase, an enzyme that is responsible for metabolism of 5-Fu. This toxicity is due to 5-Fu but not metabolites [39]. In order to bypass these resistances, FdUMP, instead of 5-Fu, was formulated using our AEAA-targeted PEGylated NP (Nano-FdUMP) (fig. 1a). Free FdUMP, being a nucleoside phosphate, is impermeable into cells [40], while Nano-FdUMP can efficiently carry the impermeable FdUMP into cancer cells (see below results).

Taken together, Nano-FdUMP potentially provides the advantages over the previously reported 5-Fu nanoformulations, from the encapsulation efficiency/loading capacity and the mechanism of action points of view.

### In vitro anticancer effects of nano-FdUMP

**Fig. 2:**
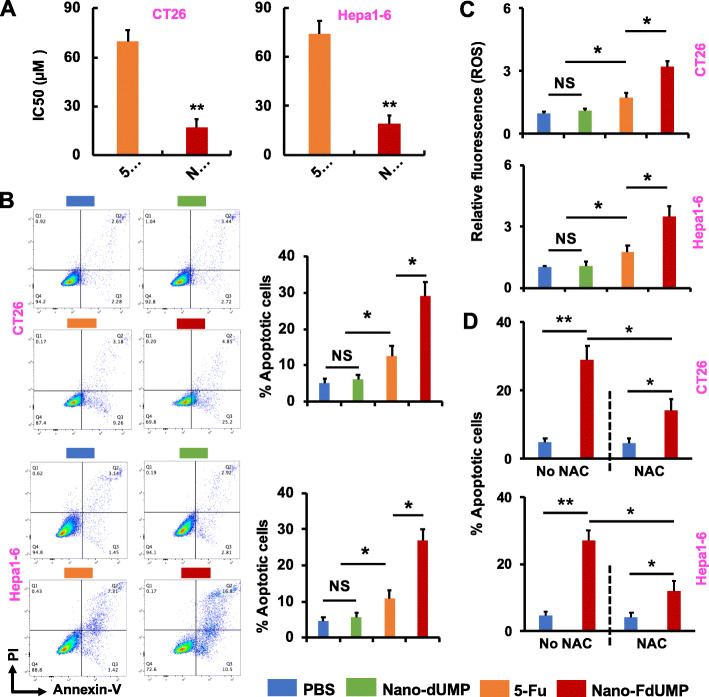
In vitro studies of Nano-FdUMP. **a**) Cytotoxicity of CT26 and Hepa1–6 cells treated with 5-Fu and Nano-FdUMP (*n* = 3, ** *p* < 0.01). **b**) Apoptotic CT26 and Hepa1–6 cells (%) treated with PBS, 5-Fu, Nano-dUMP and Nano-FdUMP were measured by Annexin V-FTIC and PI assay (n = 3, * *p* < 0.05). **c**) The ROS level in CT26 and Hepa1–6 cells treated with PBS, 5-Fu, Nano-dUMP and Nano-FdUMP (n = 3, * *p* < 0.05). **d**) Apoptotic CT26 and Hepa1–6 cells (%) treated by Nano-FdUMP following incubation with or without NAC (n = 3, * *p* < 0.05 and ** *p* < 0.01)

Nano-FdUMP caused significantly higher cytotoxicity (IC_50_ ≈ 20 μM, 24 h incubation; *p* < 0.01) in mouse CRC (CT26) and HCC (Hepa1–6) cell lines relative to 5-Fu (IC_50_ ≈ 70 μM, 24 h incubation) (fig. 2a). Nano-dUMP, in which FdUMP was replaced by 2′-deoxyuridine 5′-monophosphate (dUMP), was chosen as negative control. Of note, IC_50_ of Nano-dUMP could not be determined under the conditions tested, demonstrating that neither dUMP nor AEAA-targeted formulation was cytotoxic. In addition, no significant difference in apoptosis of CT26 and Hepa1–6 cells was observed between Nano-dUMP and PBS (fig. 2b), while Nano-FdUMP induced significantly higher level of apoptosis (*p* < 0.01, 24 h incubation) as compared to Nano-dUMP and 5-Fu (fig. 2b). These indicate that cytotoxic and apoptotic effects of Nano-FdUMP were mainly due to delivery of fluorine drug using AEAA-targeted nanoformulation.

It has been reported that the increment of ROS is associated with the progress of apoptosis [41]. The capacity of Nano-FdUMP to induce ROS was subsequently assessed in CT26 and Hepa1–6 cells (fig. 2c). Results showed that no significant difference in ROS formation was found in cancer cells between PBS and Nano-dUMP, while the ROS was significantly induced by 5-Fu (*p* < 0.05, 24 h). Furthermore, Nano-FdUMP caused significantly higher level of ROS (*p* < 0.01, 24 h) than 5-Fu (fig. 2c). Glutathione (GSH) is known as the primary endogenous antioxidant, and plays a key role in neutralization of intracellular ROS by direct and indirect scavenging [42]. As the synthesis of GSH is mainly relied on L-cysteine [43], and *N*-acetyl-L-cysteine (NAC) is the acetylated variant (a precursor) of L-cysteine [44], NAC can be used to provide L-cysteine for GSH production. Here, NAC was used to investigate the role of ROS achieved by Nano-FdUMP in the induction of apoptosis (fig. 2d). When cancer cells were pretreated with NAC, the apoptotic efficacy of Nano-FdUMP was significantly reduced (*p* < 0.05, 24 h) from ~ 30% to ~ 15% (Fig. 2d). These results showed that the apoptosis of CRC and HCC cells is, at least, in part due to ROS formation achieved by Nano-FdUMP.

### In vivo profiles of nano-FdUMP

The in vivo toxicity of Nano-FdUMP was first assessed in healthy mice (Fig. S2). No significant loss of body weight was found in Nano-FdUMP at 5, 10 and 25 mg/kg FdUMP; however, Nano-FdUMP at 50 mg/kg of FdUMP caused slight body weight loss (Fig. S2). In addition, toxic signs (e.g.*,* hunched posture, ruffled hair coat, and reluctance to move) were observed in mice treated with Nano-FdUMP at higher dose (50 mg/kg) but not at lower doses (5, 10 and 25 mg/kg) (Fig. S2).

**Fig. 3:**
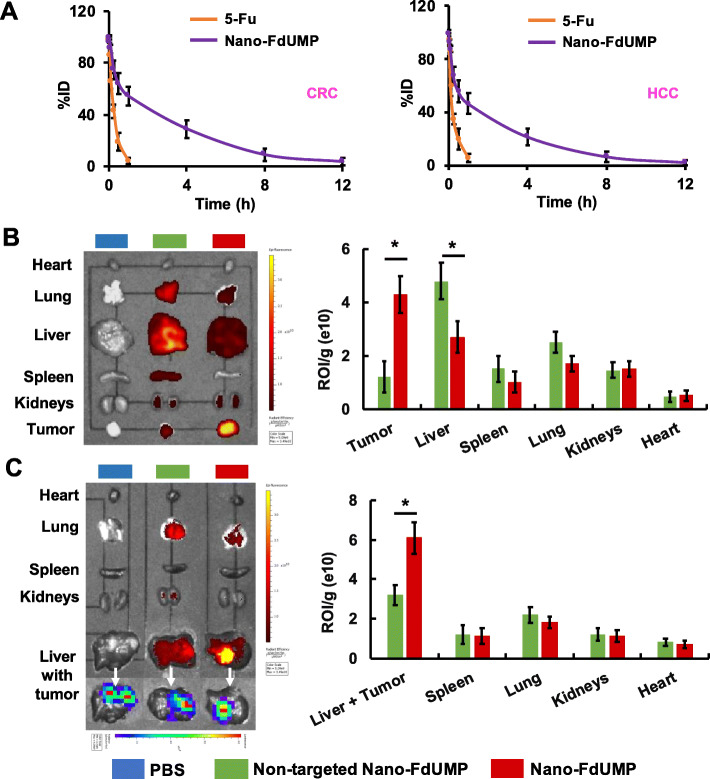
Blood circulation and biodistribution of Nano-FdUMP. 5-Fu and Nano-FdUMP were i.v. injected into orthotopic CRC and HCC mouse models. **a**) The concentration of fluorine drug on different time points was plotted (n = 4). Half-life of 5-Fu and Nano-FdUMP was assessed using a one-compartmental model. **b**) Twelve hours post i.v. administration, the distribution of Did-labeled nanoformulations into tissues and tumors was detected (640 nm/670 nm) using IVIS® Kinetics Optical System (n = 4, * *p* < 0.05) in mice grafted with CRC. **c**) Twelve hours post i.v. administration, the distribution of Did-labeled nanoformulations into tissues and tumors was detected (640 nm/670 nm) using IVIS® Kinetics Optical System (n = 4, * *p* < 0.05) in mice grafted with HCC. In HCC model, AEAA-targeted nanoformulation was specifically accumulated inside liver tumor, which was confirmed by the colocalization of NPs (fluorescent imaging from DiD dye) and tumor tissue (bioluminescence imaging from visible light produced by the interaction between luciferase and luciferin)

The i.v. administration of 5-Fu causes short blood circulation and quick systemic elimination [45]. PEGylated nanoformulation may significantly increase the half-life of chemotherapeutics in the bloodstream [46]. In this study, the half-life of Nano-FdUMP was determined using orthotopic CT26-FL3 derived CRC and Hepa1–6-Luc derived HCC mouse models (fig. 3a). Results showed that the concentration of fluorine drug in the plasma decreased rapidly, and a minor level was detected at 1 h post injection (t_1/2_ ≈ 6 min and 5 min for 5-Fu in CRC and HCC models; fig. 3a). In contrast, fluorine drug in Nano-FdUMP was more slowly eliminated from the plasma (t_1/2_ ≈ 1.6 h and 1.4 h for FdUMP in CRC and HCC models; fig. 3a). In addition, Nano-FdUMP without AEAA demonstrated similar half-lives (Fig. S3) as recorded by Nano-FdUMP with AEAA (fig. 3a). These results confirmed that the half-life of fluorine drug was significantly improved by Nano-FdUMP.

The tissue distribution of Nano-FdUMP was also investigated using orthotopic CRC and HCC mouse models. Following i.v. injection of DiD-labeled nanoformulations, tumors and major tissues were ex vivo imaged using the IVIS® Kinetics Optical System (fig. 3b and c). In CRC model, AEAA-targeted Nano-FdUMP achieved significantly higher retention in the tumor (~ 2.5 fold; *p* < 0.05) but significantly less accumulation in the liver (~ 2 fold; *p* < 0.05) than non-targeted nanoformulation (fig. 3b). In HCC model, AEAA-targeted nanoformulation was mainly accumulated inside liver tumor, which was confirmed by the colocalization of NPs (fluorescence imaging from DiD dye) and tumor tissue (bioluminescence imaging from visible light produced by luciferase in tumor cells) (fig. 3c). However, non-targeted nanoformulation was mainly found in healthy liver rather than the tumor (fig. 3c). It is known that AEAA is a high affinity ligand (Kd ≈ 9 nM) for the sigma-1 receptor which is expressed in most of solid tumor cells (e.g.*,* CT26 and Hepa1–6 cells) [7, 24]. Results in fig. 3b and c confirmed that AEAA-targeted nanoformulation significantly improved tumor accumulation and alleviated non-specific tissue distribution. Due to pH-sensitive property (fig. 1d), fluorine drug will be released from AEAA-targeted nanoformulation inside the tumor (see below discussion), which is reminiscent of Nano-Folox that could also specifically achieve the delivery and release of platinum drug and FnA inside the tumor [7].

Furthermore, the antitumor efficacy of Nano-FdUMP was assessed in orthotopic CT26-FL3 derived CRC and Hepa1–6-Luc derived HCC mouse models (Fig. S4). No significant antitumor efficacy was achieved by 5-Fu (50 mg/kg) as compared to PBS, while the growth of CRC and HCC was significantly slowed down by Nano-FdUMP (10 and 25 mg/kg) (Fig. S4), indicating that Nano-FdUMP at lower doses could achieve significantly improved therapeutic efficacy as compared to 5-Fu at higher doses. In addition, no significant antitumor efficacy was achieved by non-targeted Nano-FdUMP as compared to PBS, but AEAA-targeted Nano-FdUMP significantly retarded tumor growth (*p* < 0.05) than non-targeted nanoformulation (Fig. S5), confirming AEAA-mediated antitumor effect.

### In vitro synergistic ICD effects of nano-FdUMP and nano-Folox

It is well established that ICD-associated immunogenicity can be evoked by ROS [15], and the efficacy of ICD may be improved by ROS-inducing strategies [16–18]. It was previously reported by our laboratory that Nano-Folox resulted in OxP-mediated ICD for anticancer immune response [7]. Here, synergistic ICD effects of Nano-FdUMP and Nano-Folox were assessed using CT26 and Hepa1–6 cells in terms of ICD hallmarks, namely exposure of calreticulin (CRT), secretion of adenosine triphosphate (ATP), and release of high mobility group protein B1 (HMGB1) [15].

**Fig. 4:**
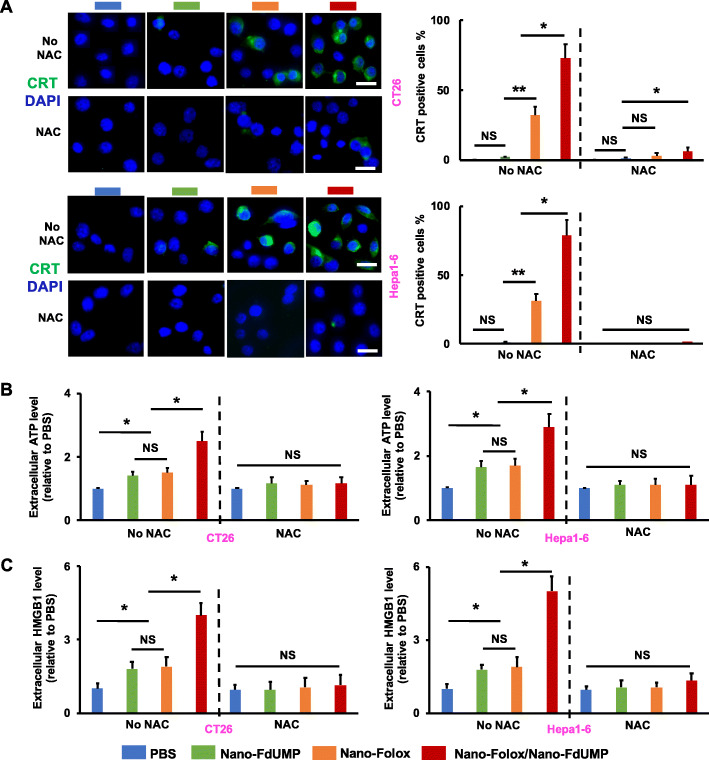
Synergistic ICD effects achieved by Nano-FdUMP and Nano-Folox. a) The exposure of CRT in CT26 and Hepa1–6 cells treated with PBS, Nano-FdUMP, Nano-Folox, and Nano-Folox/Nano-FdUMP following incubation with or without NAC (n = 3, * *p* < 0.05, ** *p* < 0.01, and no significance = NS). b) The release of ATP from CT26 and Hepa1–6 cells into the extracellular milieu treated with PBS, Nano-FdUMP, Nano-Folox, and Nano-Folox/Nano-FdUMP following incubation with or without NAC (n = 3, * *p* < 0.05). c) The secretion of HMBG1 in CT26 and Hepa1–6 cells treated with PBS, Nano-FdUMP, Nano-Folox, and Nano-Folox/Nano-FdUMP following incubation with or without NAC (n = 3, * *p* < 0.05)

Results in fig. 4a show that no significant difference in exposure of CRT was observed between Nano-FdUMP and PBS, most likely due to the inefficiency of 5-Fu or metabolites in facilitating the translocation of CRT [47]. In contrast, Nano-Folox was able to mediate significantly efficient CRT exposure (*p* < 0.01, ~ 31 to 32%) onto the cell membrane (fig. 4a), which has been previously reported [7]. Notably, combination of Nano-FdUMP and Nano-Folox further improved translocation of CRT (*p* < 0.05, ~ 73 to 79%) relative to either nanoformulations (fig. 4a).

Although 5-Fu or metabolites cannot effectively induce the CRT exposure, they may facilitate the ATP secretion and HMGB1 release [47]. Indeed, Nano-FdUMP significantly activated the secretion of ATP into the extracellular milieu of cancer cells (*p* < 0.05) as compared to PBS, which were similar to results obtained by Nano-Folox (fig. 4b). Of note, the combination of two nanoformulations further enhanced the secretion of ATP (*p* < 0.01) relative to either nanoformulations (fig. 4b).

Moreover, Nano-FdUMP significantly exerted the release of HMGB1 from the nucleus into the extracellular milieu of cancer cells as compared to PBS, which were similar to results found in Nano-Folox (fig. 4c). The combination of two nanoformulations further promoted the release of HMGB1 (*p* < 0.05) relative to either nanoformulations (fig. 4c). These results demonstrated that Nano-FdUMP was not able to induce ICD efficacy on its own, but could synergize with Nano-Folox for improved ICD effects.

It is worth noting that when cancer cells were pretreated with NAC, the activity of ICD hallmarks was significantly suppressed in either Nano-FdUMP, Nano-Folox, or combination (fig. 4), indicating that 1) the production of ROS is critical for Nano-Folox-mediated ICD induction, most likely due to the fact that OxP induces ICD via both endoplasmic reticulum (ER) stress and ROS generation; 2) the critical role of ROS achieved by Nano-FdUMP in promoting ICD effects of Nano-Folox.

### Combination of nano-FdUMP and nano-Folox for synergistic chemo-immunotherapy in orthotopic CRC and HCC mouse models

**Fig. 5:**
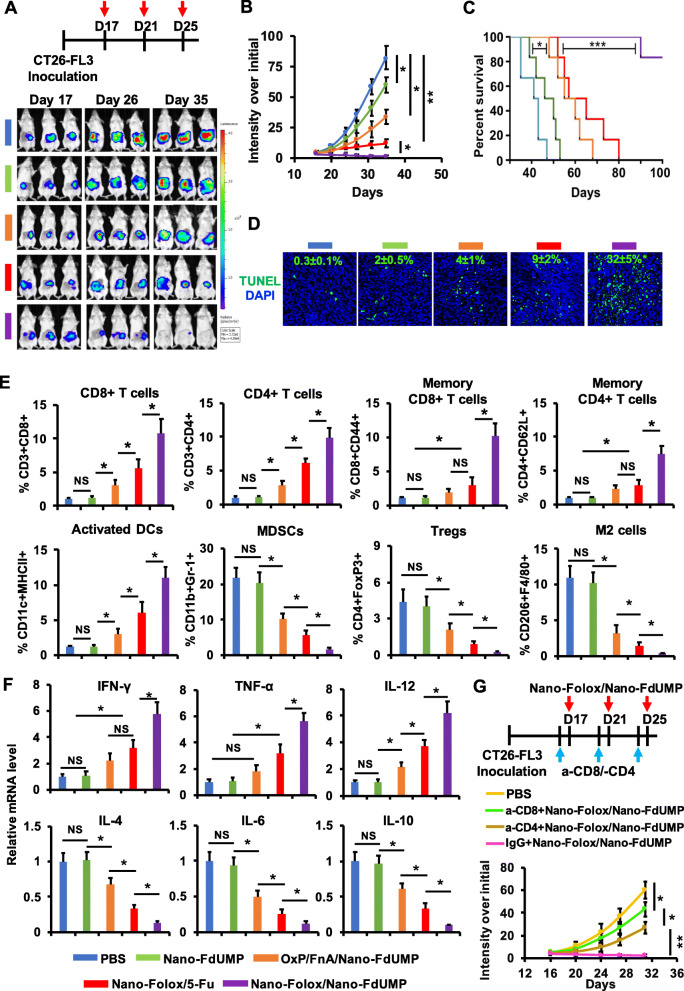
Chemo-immunotherapeutic effects of two nanoformulations in orthotopic CRC mouse model. **a**) Treatment schedule and IVIS images. **b**) The CT26-FL3 tumor growth over a 35-day period (*n* = 6, * *p* < 0.05 and ** *p* < 0.01). **c**) Animal survival (median survival: PBS = 40 days, Nano-FdUMP = 45 days, Nano-FdUMP with OxP and FnA = 58 days, and Nano-Folox with 5-Fu = 64 days) (n = 6, *** *p* < 0.001). **d**) Immunofluorescent staining of tumors on Day 24 (DNA fragments = green; nuclei = blue) to determine apoptosis (n = 4, * *p* < 0.05, relative to Nano-Folox/5-Fu). **e**) Level of CD8^+^ T cells, CD4^+^ T cells, memory CD8^+^ T cells, memory CD4^+^ T cells, activated DCs, MDSCs, Tregs and M2 cells in tumors on Day 24, analyzed by flow cytometry (n = 4, * *p* < 0.05, ** *p* < 0.01; NS = no significance). **f**) The mRNA expression of IFN-γ, TNF-α, IL-12, IL-4, IL-6 and IL-10 in tumors on Day 24 (n = 4, * *p* < 0.05). **g**) Orthotopic CT26-FL3 tumor growth treated with Nano-FdUMP/Nano-Folox after the removal of CD4^+^ or CD8^+^ T cells (n = 4, * *p* < 0.05 and ** *p* < 0.01)

**Fig. 6:**
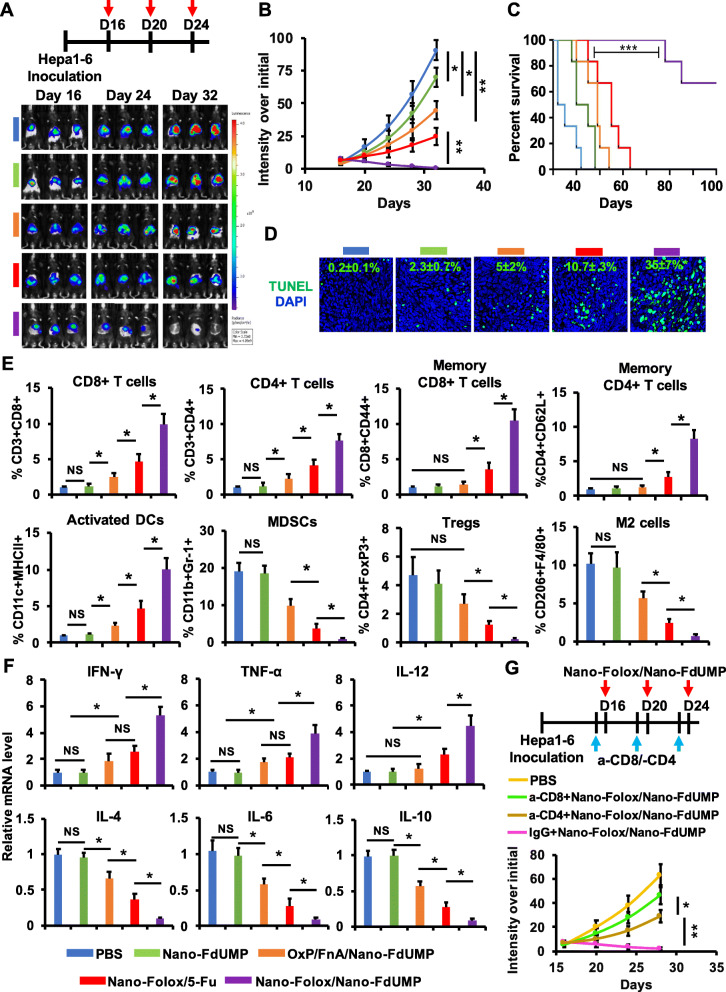
Chemo-immunotherapeutic effects of two nanoformulations in orthotopic HCC mouse model. **a**) Treatment schedule and IVIS images. **b**) The Hepa1–6-Luc tumor growth over a 32-day period (n = 6, * *p* < 0.05 and ** *p* < 0.01). **c**) Animal survival (median survival: PBS = 35 days, Nano-FdUMP = 41 days, Nano-FdUMP with OxP and FnA = 49 days, and Nano-Folox with 5-Fu = 55 days) (n = 6, *** *p* < 0.001). **d**) Immunofluorescent staining of tumors on Day 23 (DNA fragments = green; nuclei = blue) to determine apoptosis (n = 4, * *p* < 0.05, relative to Nano-Folox/5-Fu). **e**) Level of CD8^+^ T cells, CD4^+^ T cells, memory CD8^+^ T cells, memory CD4^+^ T cells, activated DCs, MDSCs, Tregs and M2 cells in tumors on Day 23, analyzed by flow cytometry (n = 4, * *p* < 0.05; NS = no significance). **f**) The mRNA expression of IFN-γ, TNF-α, IL-12, IL-4, IL-6 and IL-10 in tumors on Day 23 (n = 4, * *p* < 0.05; NS = no significance). **g**) Orthotopic Hepa1–6-Luc tumor growth treated with Nano-FdUMP/Nano-Folox after the removal of CD4^+^ or CD8^+^ T cells (n = 4, * *p* < 0.05 and ** *p* < 0.01)

Due to in vitro synergistic ICD efficacy achieved by Nano-FdUMP and Nano-Folox, the combination therapy of two nanoformulations was further evaluated in vivo using orthotopic CT26-FL3 derived CRC and Hepa1–6-Luc derived HCC mouse models (figs. 5 and 6). Based on results of Fig. S4, Nano-FdUMP containing 10 mg/kg of FdUMP was chosen for in vivo combination therapy. Based on results of fig. 3b and c and fig. S5, non-targeted Nano-FdUMP was not used for in vivo combination therapy. In addition, “Nano-Folox and free 5-Fu” has demonstrated significantly improved therapeutic outcome than FOLFOX (free drugs) [7]. Thus, “Nano-Folox and free 5-Fu” was chosen as positive control in this study.

As shown in fig. 5a and b, combination of Nano-FdUMP (10 mg/kg of FdUMP) and Nano-Folox (1.5 mg/kg of platinum drug and 4.5 mg/kg of FnA) significantly improved the antitumor efficacy than Nano-FdUMP alone, Nano-FdUMP with OxP (3 mg/kg of platinum drug) and FnA (90 mg/kg), and Nano-Folox with 5-Fu (50 mg/kg). Consequently, the combination of Nano-FdUMP and Nano-Folox provided long-term survival in 5 out of 6 mice, which was significantly improved (*p* < 0.001) than PBS [median survival (MS) = 40 days)], Nano-FdUMP (MS = 45 days), Nano-FdUMP with OxP and FnA (MS = 58 days), and Nano-Folox with 5-Fu (MS = 64 days) (fig. 5c).

Nano-Folox was reported to cause platinum-DNA-adducts for apoptosis, and the apoptotic efficacy was further enhanced when combined with 5-Fu [7]. In this study, immunofluorescence results showed that combination of Nano-FdUMP and Nano-Folox significantly (*p* < 0.05) induced apoptosis in tumors (~ 32%) relative to PBS (~ 0.3%), Nano-FdUMP alone (~ 2%), Nano-FdUMP with OxP and 5-Fu (~ 4%), and Nano-Folox with 5-Fu (~ 9%) (fig. 5d). The apoptotic efficacy was also confirmed by the detection of cleaved (activated) caspase 3 (fig. S6). The enhanced apoptotic efficacy is most likely due to the fact that 1) the efficacy of 5-Fu metabolite was promoted by FnA released from Nano-Folox; 2) 5-Fu metabolite/FnA further enhanced apoptotic effect with OxP derivative released from Nano-Folox.

The combination of two nanoformulations induced a shift from a “cold” tumor microenvironment (TME) into a “hot” one (fig. 5e and f). It was supported by the increment of immunostimulatory factors and the reduction of immunosuppressive factors. For example, CD8^+^ T cells, CD4^+^ T cells and dendritic cells (DCs) were significantly activated in tumors by the combination strategy (fig. 5e), which were accompanied with the upregulation of IFN-γ, TNF-α and IL-12, three cytokines for the activation of antitumor immunity (fig. 5f) [48]. On the contrary, myeloid derived suppressor cells (MDSCs), regulatory T cells (Tregs) and tumor-associated macrophages (M2) were significantly decreased in tumors by the combination strategy (fig. 5e), which were accompanied with downregulation of immunosuppressive cytokines such as IL-4, IL-6 and IL-10 (fig. 5f).

The ICD-associated antitumor immunity is essentially relied on the activation of effector T cells for killing tumor cells [17]. The orthotopic CRC animals were administrated with Nano-FdUMP/Nano-Folox following the depletion of either CD8^+^ or CD4^+^ T cells with corresponding monoclonal anti-CD8 or -CD4 antibody (fig. 5g). Consequently, the antitumor efficacy of Nano-FdUMP/Nano-Folox was significantly suppressed (*p* < 0.01) following the injection of these antibodies, but not the isotype IgG (fig. 5g), These results confirmed the critical role of effector T cells for antitumor immunity mediated by the combination strategy.

It has been reported that FOLFOX has demonstrated great potential for the generation of memory T cells [49], and IL-12 plays key role in the activation and proliferation of antigen-specific memory T cells [50, 51]. Indeed, memory CD8^+^ and CD4^+^ T cells were successfully activated in tumors following treatment of Nano-FdUMP/Nano-Folox (fig. 5e). In order to confirm tumor-specific memory response, tumor-free mice “cured” by the treatment of Nano-FdUMP/Nano-Folox were rechallenged with 4 T1 and CT26-FL3 cells (Fig. S7). Results showed that 4 T1 breast tumor growth was not affected, while CT26-FL3 tumor growth was significantly inhibited in same animals (Fig. S7A). These results confirmed that the combination approach promises for the induction of tumor-specific memory response against CRC, resulting in long-term survival in orthotopic CRC mice (fig. 5c).

In addition, significantly improved antitumor efficacy was also achieved by the combination strategy in orthotopic HCC mice as compared to the other controls (fig. 6a and b), which facilitated long-term survival in 4 out of 6 mice (fig. 6c). The chemo-immunotherapeutic effects including apoptosis (fig. 6d and Fig. S8) and TME remodeling (fig. 6e) were achieved by the combination strategy. The TME remodeling was supported by the increment of immunostimulatory factors and the reduction of immunosuppressive factors (fig. 6e and f). Following treatment of Nano-FdUMP/Nano-Folox, CD8^+^ T cells, CD4^+^ T cells and DCs were significantly activated in tumors (fig. 6e), which were accompanied with increase of IFN-γ, TNF-α and IL-12 (fig. 6f). In contrast, MDSCs, Tregs and M2 cells were significantly decreased in tumors (fig. 6e), which were accompanied with alleviation of IL-4, IL-6 and IL-10 (fig. 6f). In addition, the antitumor efficacy of Nano-FdUMP/Nano-Folox was also significantly suppressed in HCC mouse model following the pretreatment of anti-CD8 or anti-CD4 antibodies (fig. 6g), confirming the critical roles of effector T cells for antitumor immunity mediated by the combination strategy. Furthermore, tumor-free mice “cured” by the combined approach were rechallenged with B16 melanoma and Hepa1–6-Luc cells (Fig. S7B). Results showed that B16 tumor growth was not affected in cured mice, while Hepa1–6-Luc tumor growth was significantly suppressed in same animals (Fig. S7B). These results showed that the combination approach also has the potential for the induction of tumor-specific memory response against HCC, facilitating long-term survival in mice (fig. 6c).

In addition, no toxic signs were caused by the combination strategy as compared to PBS, which was confirmed by the analysis of body weight, hematological toxicity, and liver/kidney damage in healthy mice (Fig. S9). Taken together, the “Nano-FdUMP + Nano-Folox” strategy could achieve synergistic chemo-immunotherapeutic efficacy against CRC and HCC for long-term animal survival, without causing significant side effects.

### Blockade of PD-L1 enhanced combination of nano-FdUMP and nano-Folox for inhibition of liver metastasis

**Fig. 7:**
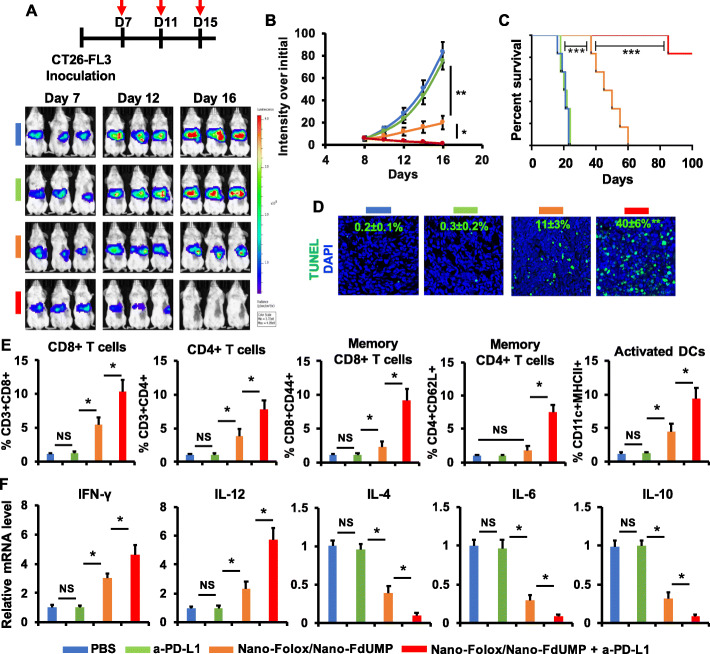
The combination therapy of Nano-FdUMP/Nano-Folox and anti-PD-L1 antibody for CRC liver metastasis mouse model. **a**) Treatment schedule and IVIS images. **b**) The liver metastases over a 16-day period (n = 6, * *p* < 0.05 and ** *p* < 0.01). **c**) Animal survival (median survival: PBS = 20 days, anti-PD-L1 antibody = 21 days, and Nano-FdUMP/Nano-Folox = 48 days) (n = 6, *** *p* < 0.01). **d**) Immunofluorescent staining of tumors on Day 12 (DNA fragments = green; nuclei = blue) to determine apoptosis (n = 4, ** *p* < 0.01, relative to Nano-FdUMP/Nano-Folox). **e**) Level of CD8^+^ T cells, CD4^+^ T cells, memory CD8^+^ T cells, memory CD4^+^ T cells, and activated DCs in tumors on Day 12, analyzed by flow cytometry (n = 4, * *p* < 0.05 and NS = no significance). **f**) The mRNA expression of IFN-γ, IL-12, IL-4, IL-6 and IL-10 in tumors on Day 12 (n = 4, * *p* < 0.05 and NS = no significance)

FOLFOX has been used for patients with unresectable CRC liver metastases [3]; however, therapeutic outcome is still poor due to fast tumor progression. In this study, the “Nano-FdUMP + Nano-Folox” strategy was further applied to treat mice with experimental liver metastasis (fig. 7). This tumor-bearing model closely reproduces the aggressive pattern of CRC at metastatic stage [52]. As shown in fig. 7a and b, the combined approach was able to significantly (*p* < 0.01) slow down tumor growth in mice as compared to PBS, which was accompanied by apoptosis (fig. 7 d and Fig. S10). In addition, Nano-FdUMP/Nano-Folox significantly reprogrammed the TME in liver metastatic site (fig. 7e and f). However, no long-term survival (MS = 48 days) was achieved by the Nano-FdUMP/Nano-Folox strategy after dosing (fig. 7c).

When combined with anti-PD-L1 mAb, Nano-FdUMP/Nano-Folox significantly inhibited liver metastases (*p* < 0.01) as compared to either Nano-FdUMP/Nano-Folox or anti-PD-L1 mAb (fig. 7a and b), which was accompanied with improved apoptosis (fig. 7d and Fig. S10). Of note, combination of Nano-FdUMP/Nano-Folox and anti-PD-L1 mAb was able to provide long-term survival in 5 out of 6 mice (fig. 7c). It is most likely due to the fact that combination of Nano-FdUMP/Nano-Folox and anti-PD-L1 mAb significantly (*p* < 0.05 and *p* < 0.01) increased the amount of effector/memory T cells and DCs (fig. 7e), upregulated the expression of IFN-γ and IL-12 (fig. 7f) and reduced the level of IL-4, IL-6, and IL-10 (fig. 7f) in liver metastatic site, as compared to either FdUMP/Nano-Folox or anti-PD-L1 mAb. These indicated that FdUMP/Nano-Folox may significantly remodel the immunosuppressive TME for enhanced antitumor outcome in combination with immune checkpoint blockade, potentially providing a chemo-immunotherapeutic strategy for metastatic CRC.

## Discussion

The development of nano delivery systems has significantly improved therapeutic efficacy and reduced side effects of anticancer agents [53]. However, the number of cancer nanomedicines approved for patients is still very few. Recently, four strategic directions have been proposed to foster nanomedicine translation and exploitation, including rational drug selection, combination therapy, immunotherapy, and patient stratification [54]. Our combination approach fulfills these directions, as follows:

1) Rational drug selection for chemo-immunotherapy. FOLFOX has been long utilized as the standard chemotherapy for CRC and HCC patients at advanced stages [1, 2]. It also demonstrates great potential for induction of robust antigen release from dying tumor cells and for generation of effector and memory T cells [55, 56]. In this study, two AEAA-targeted PEGylated nanoformulations namely Nano-FdUMP (fig. 1) and Nano-Folox [7] were applied for FOLFOX-associated CRC and HCC therapies. Nano-FdUMP induced the formation of ROS (fig. 2), which significantly promoted Nano-Folox-mediated ICD efficacy (fig. 4). Consequently, synergistic chemo-immunotherapeutic efficacy was achieved in both orthotopic CRC and HCC mouse models using the “Nano-FdUMP + Nano-Folox” strategy (figs. 5 and 6).

2) Combination therapy in microsatellite stable (MSS) CRC patients. Immune checkpoint inhibitors (e.g.*,* anti-PD-L1 mAb) have demonstrated efficacy in different cancers, but the response rate is still poor in CRC patients. Only a minor population of patients, who are diagnosed with microsatellite instable (MSI) CRC (~ 15% of total population) [57], respond to anti-PD-L1 mAb as a monotherapy [58]. It is now known that the immunosuppressive TME (also characterized as “cold” tumor) causes inefficiency of immune checkpoint inhibitors [59, 60]. The shift of “cold” tumor to “hot” one potentially enhances the efficacy of checkpoint blockade [61]. In this study, the “Nano-FdUMP + Nano-Folox” strategy was able to induce ICD-associated antitumor immunity, which significantly reprogrammed immunosuppressive TME, improving antitumor efficacy against MSS CRC liver metastasis (established by CT26-FL3 cells, an MSS CRC cell line [62, 63]) in combination with anti-PD-L1 mAb (fig. 7). Therefore, the combination of Nano-FdUMP/Nano-Folox and anti-PD-L1 mAb will potentially achieve a superior outcome for CRC patients (particularly for MSS ones, up to 85% of total population) at advanced stages.

It is theoretically possible to encapsulate OxP, FnA and 5-Fu in a single nanoformulation. However, in comparison with the “all in one” nanoformulation, the “Nano-FdUMP + Nano-Folox” strategy holds greater potential for clinical translation, as follows:

1) Development of complex nanomedicines may not be beneficial for translation into clinical use [64, 65]. The “all in one” nanoformulation achieved using distinct functional materials may complicate large-scale manufacturing and cause unwanted toxic effects. In this study, Nano-Folox and Nano-FdUMP were achieved using nanoprecipitation process. It is a well-established formulation technique with biodegradable and biocompatible materials, and has been substantially utilized in our laboratory for delivery of chemotherapeutics and nucleic acids [21–23, 28, 30–34].

2) A variety of FOLFOX regimens are available in clinic such as FOLFOX-4, FOLFOX-6 and FOLFOX-7, and they differ in dose schedule of OxP and 5-Fu, and are chosen for cancer patients at different stages [66]. Our “Nano-FdUMP + Nano-Folox” strategy can be adjusted according to the clinical practice, while the adjustment in dose and timing of OxP and 5-Fu is difficult in an “all in one” nanoformulation.

## Conclusions

This study provides proof of concept that combination of two nano delivery systems may overcome the barriers associated with FOLFOX including non-specific delivery, high toxicity and long course of treatment, which can result in successful treatment for CRC and HCC. Further optimization in terms of dosing and timing will enhance clinical potential of our combination strategy for patients.

## Supplementary Information

**Additional file 1 Fig. S1.** The physicochemical characterization of non-targeted Nano-FdUMP. A) TEM image (bar = 100 nm). B) Size distribution (~ 38 nm, polydispersity index ≈ 0.3) and surface charge (~ 5 mV). C) The in vitro release of fluorine drug from nanoprecipitates in pH = 5.5 and 7.4 (*n* = 4). d) No significant aggregation was caused in 10% serum-containing medium up to 12 h at 37 °C. **Fig. S2**. Toxicity of Nano-FdUMP in healthy BALB/C mice. A) The body weight over a 35-day period following treatment of PBS and Nano-FdUMP containing 5, 10, 25 and 50 mg/kg FdUMP on Day 1, 3 and 5. B) The overall condition of animals (*n* = 5) based on body condition scoring [BCS, IACUC Guidelines along with other criteria (e.g., hunched posture, ruffled hair coat, and reluctance to move)]. At the endpoint, the number of animals compliant with BCS index was presented. Results of non-targeted Nano-FdUMP were similar to those observed in targeted counterpart (Data not shown). **Fig. S3.** Blood circulation of non-targeted Nano-FdUMP in orthotopic CRC and HCC mouse models. Following i.v. injection, the concentration of fluorine drug on different time points was plotted (n = 4). Results showed that non-targeted Nano-FdUMP demonstrated similar blood circulation recorded by targeted counterpart (Fig. 3a). **Fig. S4**. Therapeutic efficacy of Nano-FdUMP in orthotopic CRC and HCC mouse models. Following treatment schedule as described in Figs. 5 and 6, Nano-FdUMP at doses of 10 and 25 mg/kg FdUMP achieved significantly improved antitumor efficacy as compared to PBS and 5-Fu at 50 mg/kg (n = 5, * *p* < 0.05 and *p* < 0.01). **Fig. S5**. Therapeutic efficacy of Nano-FdUMP with/without AEAA at dose of 10 mg/kg FdUMP in orthotopic CRC and HCC mouse models. Following treatment schedule as described in Figs. 5 and 6, non-targeted Nano-FdUMP could not slow down tumor growth as compared to PBS, but AEAA-targeted Nano-FdUMP achieved significantly improved antitumor efficacy than PBS and non-targeted Nano-FdUMP (n = 5, * *p* < 0.05). **Fig. S6.** Immunofluorescent staining of tumors on Day 24 (as described in Fig. 5) (cleaved caspase 3 = green; nuclei = blue) to determine apoptosis (*n* = 3, ** *p* < 0.01, relative to Nano-Folox/5-Fu). **Fig. S7**. Rechallenge studies. A) Orthotopic CRC mice (BALB/C) were treated with Nano-FdUMP/Nano-Folox as described in Fig. 5, and one month after tumor disappearance, two flanks of mice were respectively rechallenged with 4 T1 and CT26-FL3 cells (1 × 10^6^ cells per mouse) (n = 4). B) Orthotopic HCC mice (C57BL/6) were treated with Nano-FdUMP/Nano-Folox as described in Fig. 6, and one month after tumor disappearance, two flanks of mice were respectively rechallenged with B16 and Hepa1–6-Luc cells (1 × 10^6^ cells per mouse) (n = 4). Tumor volume was calculated using the formula a^2^b(π/6), where a is the minor diameter of the tumor and b is the major diameter perpendicular to diameter a. Results confirmed that Nano-FdUMP/Nano-Folox could induce tumor-specific memory response. **Fig. S8**. Immunofluorescent staining of tumors on Day 23 (as described in Fig. 6) (cleaved caspase 3 = green; nuclei = blue) to determine apoptosis (n = 3, ** *p* < 0.01, relative to Nano-Folox/5-Fu). **Fig. S9**. Toxicity studies of two nanoformulations in A) BALB/C and B) C57BL/6 mice. The body weight over a 35-day period following treatment of PBS and combination of two nanoformulations (Nano-Folox containing 1.5 mg/kg platinum drug was i.v. injected into mice on Day 1, 3 and 5. Eight hours post injection, Nano-FdUMP containing 10 mg/kg fluorine drug was i.v. injected into mice). Results show that no significant change was found in body weight and hematological/liver/kidney functions following treatment of two nanoformulations as compared to PBS (n = 5). **Fig. S10**. Immunofluorescent staining of tumors on Day 12 (as described in Fig. 7) (cleaved caspase 3 = green; nuclei = blue) to determine apoptosis (n = 3, ** *p* < 0.01, relative to Nano-FdUMP/Nano-Folox). **Table S1**. Antibodies used in the study. **Table S2**. Primers used for RT-PCR in the study.

## Data Availability

All data generated during this study are included in this published article and its supplementary files.

## Abbreviations

AEAA: Aminoethyl anisamide
ATP: Adenosine triphosphate
CRC: Colorectal cancer
CRT: Calreticulin
DAMPs: Damage-associated molecular patterns
DCs: Dendritic cells
DOPA: 1,2-dioleoyl-*sn*-glycero-3-phosphate
DOTAP: 1,2-dioleoyl-3-trimethylammonium-propane
DSPE-PEG: N-(Carbonyl-methoxypolyethyleneglycol 2000)-1,2-distearoyl-sn-glycero-3-phosphoethanolamine
dTMP: Deoxythymidine monophosphate
dUMP: 2′-deoxyuridine 5′-monophosphate (dUMP)
FdUMP: 5-Fluoro-2′-deoxyuridine 5′-monophosphate (FdUMP)
FnA: Folinic acid
GSH: Glutathione
HCC: Hepatocellular carcinoma
HMGB1: High mobility group protein B1
ICD: Immunogenic cell death
mAb: Monoclonal antibody
MDSCs: Myeloid derived suppressor cells (MDSCs)
NAC: *N*-acetyl-L-cysteine
NP: Nanoparticle
OxP: Oxaliplatin
PEG: Polyethylene glycol
PFA: Paraformaldehyde
ROS: Reactive oxygen species
TEM: Transmission electron microscope
TME: Tumor microenvironment
Tregs: Regulatory T cells
5-Fu: Fluorouracil
