# Supplementary material for: Two nanoformulations induce reactive oxygen species and immunogenetic cell death for synergistic chemo-immunotherapy eradicating colorectal cancer and hepatocellular carcinoma
Source: Mol Cancer. 2021 Jan 6;20:10. doi: 10.1186/s12943-020-01297-0 (PMC7786897; doi:10.1186/s12943-020-01297-0)
Supplement: Supplementary file 1 — Additional file 1 Fig. S1. The physicochemical characterization of non-targeted Nano-FdUMP. A) TEM image (bar = 100 nm). B) Size distribution (~ 38 nm, polydispersity index ≈ 0.3) and surface charge (~ 5 mV). C) The in vitro release of fluorine drug from nanoprecipitates in pH = 5.5 and 7.4 (n = 4). d) No significant aggregation was caused in 10% serum-containing medium up to 12 h at 37 °C. Fig. S2. Toxicity of Nano-FdUMP in healthy BALB/C mice. A) The body weight over a 35-day period following treatment of PBS and Nano-FdUMP containing 5, 10, 25 and 50 mg/kg FdUMP on Day 1, 3 and 5. B) The overall condition of animals (n = 5) based on body condition scoring [BCS, IACUC Guidelines along with other criteria (e.g., hunched posture, ruffled hair coat, and reluctance to move)]. At the endpoint, the number of animals compliant with BCS index was presented. Results of non-targeted Nano-FdUMP were similar to those observed in targeted counterpart (Data not shown). Fig. S3. Blood circulation of non-targeted Nano-FdUMP in orthotopic CRC and HCC mouse models. Following i.v. injection, the concentration of fluorine drug on different time points was plotted (n = 4). Results showed that non-targeted Nano-FdUMP demonstrated similar blood circulation recorded by targeted counterpart (Fig. 3a). Fig. S4. Therapeutic efficacy of Nano-FdUMP in orthotopic CRC and HCC mouse models. Following treatment schedule as described in Figs. 5 and 6, Nano-FdUMP at doses of 10 and 25 mg/kg FdUMP achieved significantly improved antitumor efficacy as compared to PBS and 5-Fu at 50 mg/kg (n = 5, * p < 0.05 and p < 0.01). Fig. S5. Therapeutic efficacy of Nano-FdUMP with/without AEAA at dose of 10 mg/kg FdUMP in orthotopic CRC and HCC mouse models. Following treatment schedule as described in Figs. 5 and 6, non-targeted Nano-FdUMP could not slow down tumor growth as compared to PBS, but AEAA-targeted Nano-FdUMP achieved significantly improved antitumor efficacy than PBS and non-targeted Nano-FdUMP (n = [file 12943_2020_1297_MOESM1_ESM.docx]

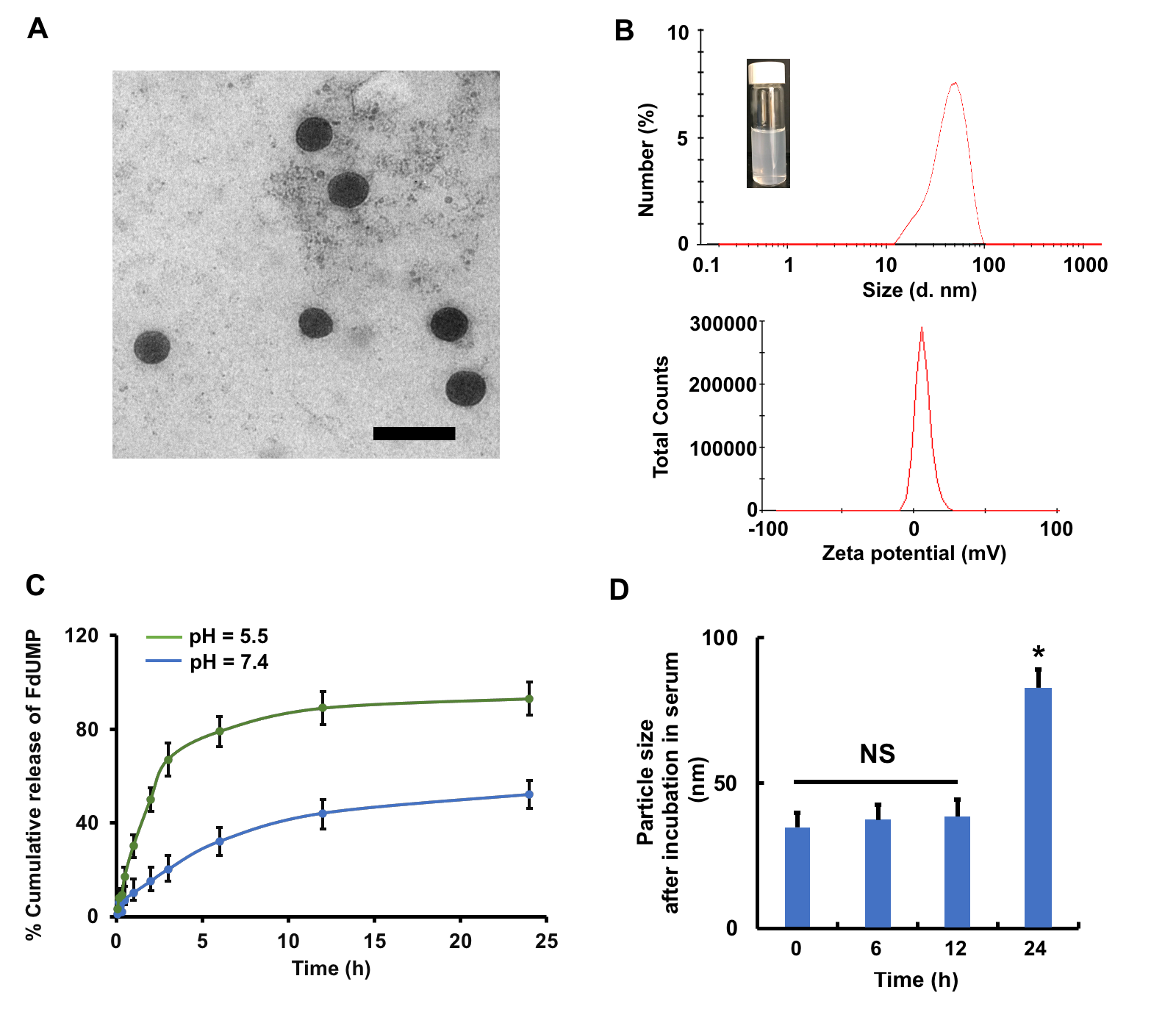


**Figure S1.** The physicochemical characterization of non-targeted Nano-FdUMP. A) TEM image (bar = 100 nm). B) Size distribution (~ 38 nm, polydispersity index ≈ 0.3) and surface charge (~ 5 mV). C) The *in vitro* release of fluorine drug from nanoprecipitates in pH = 5.5 and 7.4 (n = 4). d) No significant aggregation was caused in 10% serum-containing medium up to 12 h at 37 °C.

**Figure S2**. Toxicity of Nano-FdUMP in healthy BALB/C mice. A) The body weight over a 35-day period following treatment of PBS and Nano-FdUMP containing 5, 10, 25 and 50 mg/kg FdUMP on Day 1, 3 and 5. B) The overall condition of animals (n = 5) based on body condition scoring [BCS, IACUC Guidelines along with other criteria (e.g., hunched posture, ruffled hair coat, and reluctance to move)]. At the endpoint, the number of animals compliant with BCS index was presented. Results of non-targeted Nano-FdUMP were similar to those observed in targeted counterpart (Data not shown).


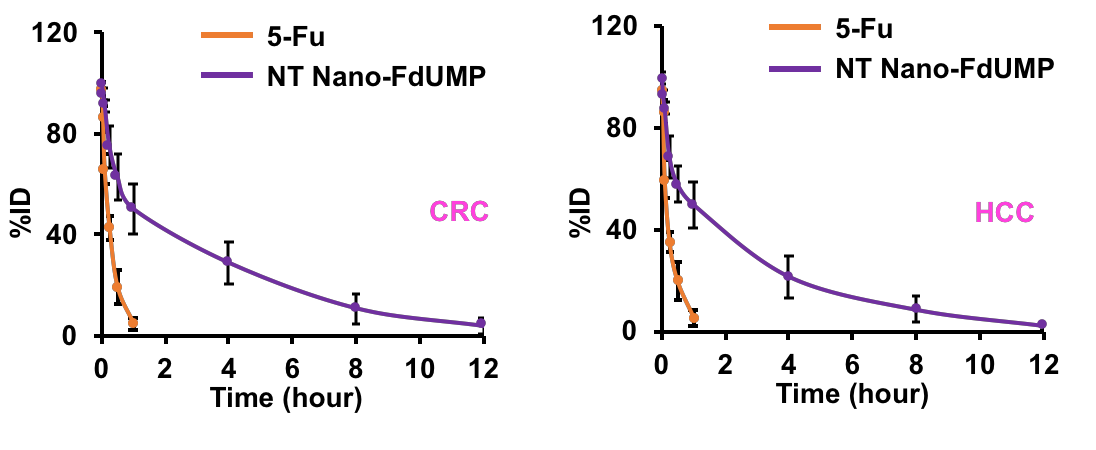


**Figure S3.** Blood circulation of non-targeted Nano-FdUMP in orthotopic CRC and HCC mouse models. Following i.v. injection, the concentration of fluorine drug on different time points was plotted (n = 4). Results showed that non-targeted Nano-FdUMP demonstrated similar blood circulation recorded by targeted counterpart (Figure 3a).


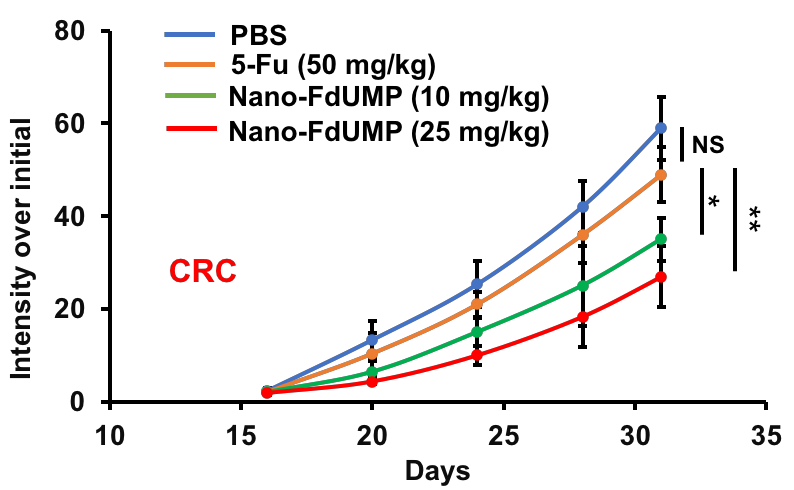

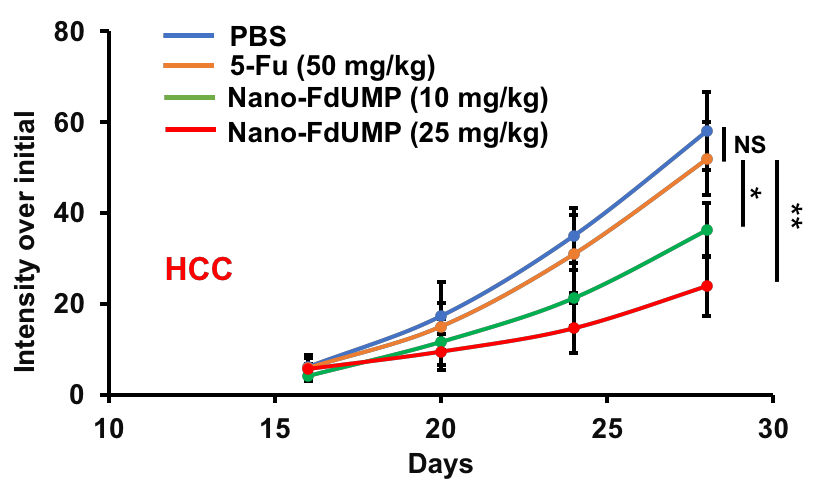


**Figure S4**. Therapeutic efficacy of Nano-FdUMP in orthotopic CRC and HCC mouse models. Following treatment schedule as described in Figures 5 and 6, Nano-FdUMP at doses of 10 and 25 mg/kg FdUMP achieved significantly improved antitumor efficacy as compared to PBS and 5-Fu at 50 mg/kg (n = 5, * *p* < 0.05 and *p* < 0.01).

**Figure S5**. Therapeutic efficacy of Nano-FdUMP with/without AEAA at dose of 10 mg/kg FdUMP in orthotopic CRC and HCC mouse models. Following treatment schedule as described in Figures 5 and 6, non-targeted Nano-FdUMP could not slow down tumor growth as compared to PBS, but AEAA-targeted Nano-FdUMP achieved significantly improved antitumor efficacy than PBS and non-targeted Nano-FdUMP (n = 5, * *p* < 0.05).

**
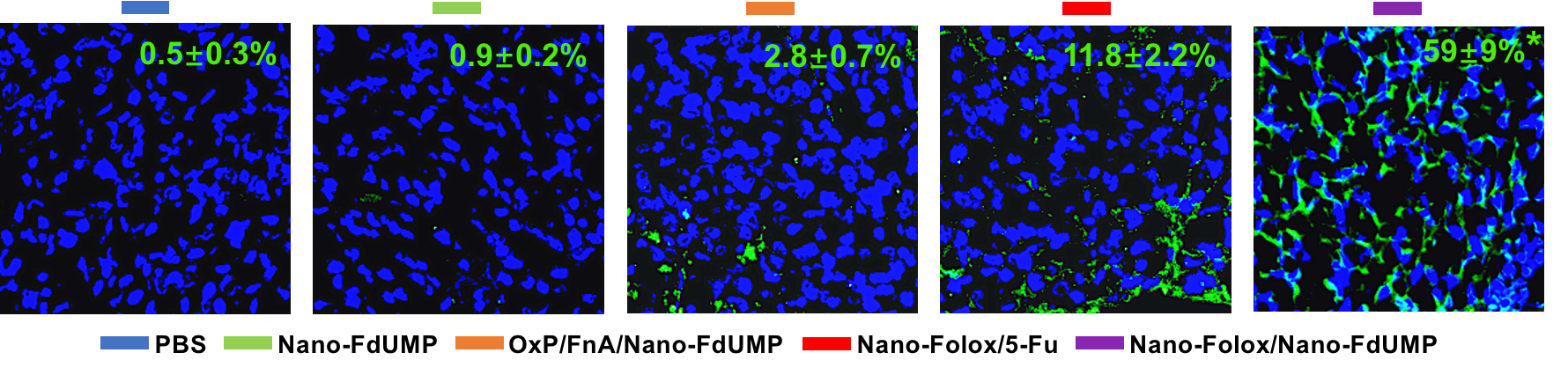
**

**Figure S6.** Immunofluorescent staining of tumors on Day 24 (as described in Figure 5) (cleaved caspase 3 = green; nuclei = blue) to determine apoptosis (n = 3, ** *p* < 0.01, relative to Nano-Folox/5-Fu).

**Figure S7**. Rechallenge studies. A) Orthotopic CRC mice (BALB/C) were treated with Nano-FdUMP/Nano-Folox as described in Figure 5, and one month after tumor disappearance, two flanks of mice were respectively rechallenged with 4T1 and CT26-FL3 cells (1 x 10^6^ cells per mouse) (n = 4). B) Orthotopic HCC mice (C57BL/6) were treated with Nano-FdUMP/Nano-Folox as described in Figure 6, and one month after tumor disappearance, two flanks of mice were respectively rechallenged with B16 and Hepa1-6-Luc cells (1 x 10^6^ cells per mouse) (n = 4). Tumor volume was calculated using the formula a^2^b(π/6), where a is the minor diameter of the tumor and b is the major diameter perpendicular to diameter a. Results confirmed that Nano-FdUMP/Nano-Folox could induce tumor-specific memory response.


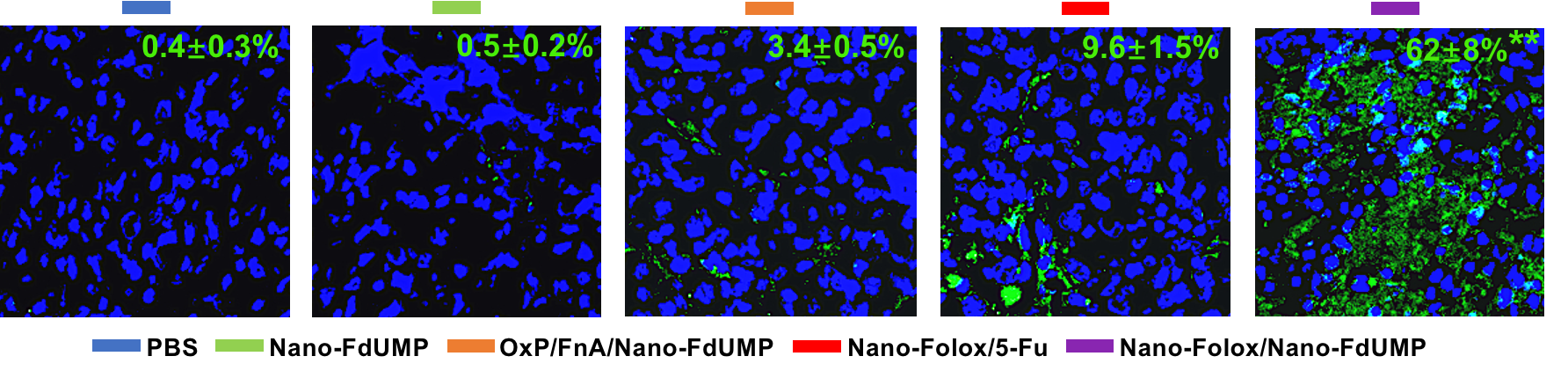


**Figure S8**. Immunofluorescent staining of tumors on Day 23 (as described in Figure 6) (cleaved caspase 3 = green; nuclei = blue) to determine apoptosis (n = 3, ** *p* < 0.01, relative to Nano-Folox/5-Fu).

**Figure S9**. Toxicity studies of two nanoformulations in A) BALB/C and B) C57BL/6 mice. The body weight over a 35-day period following treatment of PBS and combination of two nanoformulations (Nano-Folox containing 1.5 mg/kg platinum drug was i.v. injected into mice on Day 1, 3 and 5. Eight hours post injection, Nano-FdUMP containing 10 mg/kg fluorine drug was i.v. injected into mice). Results show that no significant change was found in body weight and hematological/liver/kidney functions following treatment of two nanoformulations as compared to PBS (n = 5).


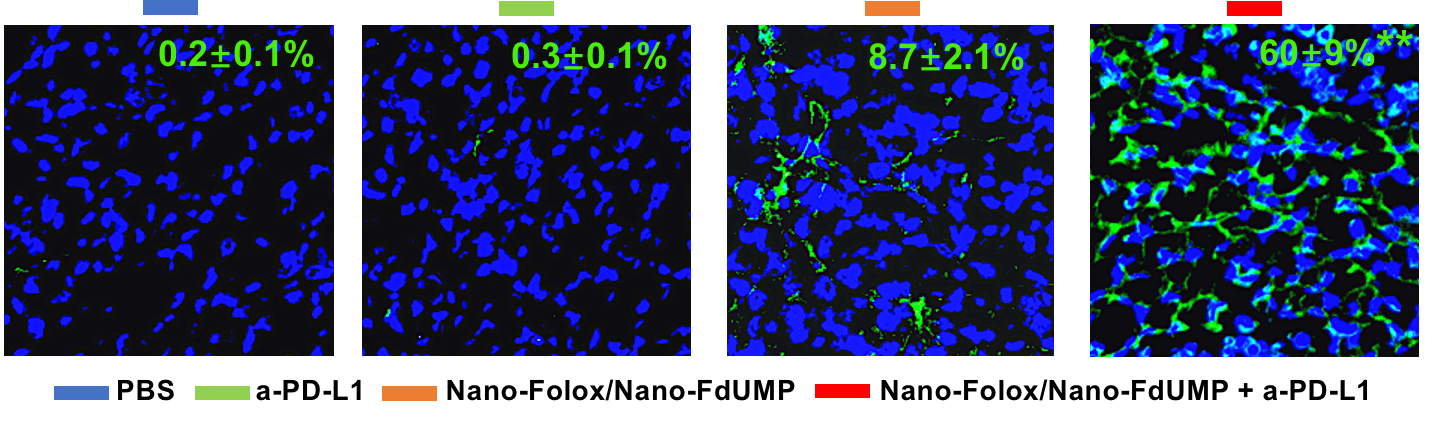


**Figure S10**. Immunofluorescent staining of tumors on Day 12 (as described in Figure 7) (cleaved caspase 3 = green; nuclei = blue) to determine apoptosis (n = 3, ** *p* < 0.01, relative to Nano-FdUMP/Nano-Folox).

**Table S1**. Antibodies used in the study.

| **Antibody** | **Company** | **Catalog No.** | **Experiment** | **Dilution** |
| --- | --- | --- | --- | --- |
| Alexa Fluor® 700 Anti-CD8 | BD Bioscience | 557959 | Flow | 1:500 |
| Alexa Fluor®647 Anti-CD3 | BioLegend | 100209 | Flow | 1:500 |
| PE Anti-CD3 | BioLegend | 100219 | Flow | 1:500 |
| FITC Anti-CD4 | BioLegend | 100405 | Flow | 1:500 |
| APC Anti-CD4 | BioLegend | 100411 | Flow | 1:500 |
| FITC Anti-CD44 | BioLegend | 103005 | Flow | 1:500 |
| APC Anti-CD62L | eBioscinece | 17-0621-81 | Flow | 1:500 |
| FITC Anti-CD11c | BioLegend | 117305 | Flow | 1:500 |
| Alexa Fluor®647 Anti-MHC II | BioLegend | 107617 | Flow | 1:500 |
| Alexa Fluor®488 CD11b | BioLegend | 101217 | Flow | 1:500 |
| APC Anti-Gr1 | BioLegend | 108412 | Flow | 1:500 |
| PE Anti-CD206 | BioLegend | 141705 | Flow | 1:500 |
| Alexa Fluor®647 Anti-F4/80 | BioLegend | 123121 | Flow | 1:500 |
| Alexa Fluor®488 FoxP3 | BioLegend | 126406 | Flow | 1:500 |
| Anti-cleaved caspase-3 antibody | abcam | ab214430 | IF | 1:500 |
| Goat anti-Rabbit IgG (Alexa Fluor 488) | abcam | Ab150077 | IF | 1:500 |

**Table S2**. Primers used for RT-PCR in the study.

| Primer | Catalog No.  (Applied Biosystems) |
| --- | --- |
| TNF-α | Mm00443260_g1 |
| IFN-γ | Mm01168134_m1 |
| IL-4 | Mm00445259_m1 |
| IL-6 | Mm00446190_m1 |
| IL-10 | Mm01288386_m1 |
| IL-12 | Mm00434169_m1 |
| GAPDH | Mm99999915_g1 |
